# Theranostic Interpolation of Genomic Instability in Breast Cancer

**DOI:** 10.3390/ijms23031861

**Published:** 2022-02-07

**Authors:** Rabia Rasool, Inam Ullah, Bismillah Mubeen, Sultan Alshehri, Syed Sarim Imam, Mohammed M. Ghoneim, Sami I. Alzarea, Fahad A. Al-Abbasi, Bibi Nazia Murtaza, Imran Kazmi, Muhammad Shahid Nadeem

**Affiliations:** 1 Institute of Molecular Biology and Biotechnology, The University of Lahore, Lahore 54000, Pakistan; rabia.amjad545499@gmail.com (R.R.); inamgenetics@gmail.com (I.U.); bismillah.mubeen@gmail.com (B.M.); 2 Department of Pharmaceutics, College of Pharmacy, King Saud University, Riyadh 11451, Saudi Arabia; salshehri1@ksu.edu.sa (S.A.); simam@ksu.edu.sa (S.S.I.); 3 Department of Pharmacy Practice, College of Pharmacy, AlMaarefa University, Ad Diriyah 13713, Saudi Arabia; mghoneim@mcst.edu.sa; 4 Department of Pharmacology, College of Pharmacy, Jouf University, Sakaka 72341, Saudi Arabia; samisz@ju.edu.sa; 5 Department of Biochemistry, Faculty of Science, King Abdulaziz University, Jeddah 21589, Saudi Arabia; fabbasi@kau.edu.sa; 6 Department of Zoology, Abbottabad University of Science and Technology (AUST), Abbottabad 22310, Pakistan; nazia.murtaza@gmail.com

**Keywords:** breast cancer, genomic instability, DNA repair pathways, PARP inhibitor

## Abstract

Breast cancer is a diverse disease caused by mutations in multiple genes accompanying epigenetic aberrations of hazardous genes and protein pathways, which distress tumor-suppressor genes and the expression of oncogenes. Alteration in any of the several physiological mechanisms such as cell cycle checkpoints, DNA repair machinery, mitotic checkpoints, and telomere maintenance results in genomic instability. Theranostic has the potential to foretell and estimate therapy response, contributing a valuable opportunity to modify the ongoing treatments and has developed new treatment strategies in a personalized manner. “Omics” technologies play a key role while studying genomic instability in breast cancer, and broadly include various aspects of proteomics, genomics, metabolomics, and tumor grading. Certain computational techniques have been designed to facilitate the early diagnosis of cancer and predict disease-specific therapies, which can produce many effective results. Several diverse tools are used to investigate genomic instability and underlying mechanisms. The current review aimed to explore the genomic landscape, tumor heterogeneity, and possible mechanisms of genomic instability involved in initiating breast cancer. We also discuss the implications of computational biology regarding mutational and pathway analyses, identification of prognostic markers, and the development of strategies for precision medicine. We also review different technologies required for the investigation of genomic instability in breast cancer cells, including recent therapeutic and preventive advances in breast cancer.

## 1. Introduction

Breast cancer occurs due to the abnormal functioning of genes controlling the growth and differentiation of cells, which may be caused by any pathological processes or environmental exposure [1]. The female population is 49.5% of the entire world’s population, out of which a large portion is females that are more than 60 years of age. A total of 2.3 million women were diagnosed with breast cancer by 2020, and 685,050 women will lose their lives to the disease. There were 7.8 million women who had been diagnosed with breast cancer in the past five years as of the end of 2020, making it the most common cancer in the world. Breast cancer is the leading cause of disability-adjusted life years (DALYs) lost in women worldwide. Breast cancer affects women of all ages after puberty; however, the incidence rises with age [2,3]. In the United States, approximately 160,000 patients suffered from advanced breast cancer [4]. Despite the fact that developed countries have the greatest incidence rates, Asia and Africa accounted for 63% of all deaths in 2020 [5]. Women in high-income nations are more likely to survive breast cancer than those in low- and middle-income countries [6]. Any genomic aberration may selectively deliberate the clones of cells via promoting their outgrowth, ultimately governing their native tissue environment. Moreover, some additional factors such as surrounding stroma, the metabolic and hormonal milieu, and the immune system play a significant role in directing clones’ behavior. The hallmark of cancer includes the acquired functional capabilities of cancer cells that promote their survival, proliferation, and dissemination, which favors tumor growth and metastatic process [7]. The breast cancer etiology and signaling cascades involved in its proliferation are the main emerging research areas during the past decades. Germline BRCA testing is performed as diagnostic in metastatic breast cancer patients, given the availability of PARP (poly ADP-ribose polymerase) inhibitors [8,9,10]. The inherited breast cancer risk is, to a lesser degree, associated with several syndromes interrelated to germline mutations of DNA repair and genomic integrity-maintaining genes. ATM, PALB2, CHEK2, PTEN, TP53, and STK11 are a new panel of genes discovered via next-generation sequencing beyond BRCA1 and BRCA2 to assess inherited breast cancer [11]. The proposed mechanisms include the change in mammary gland sensitivity to later exposure of hormones, the amount of stem cells or progenitor cell reduction that consequently causes the elimination of the target for malignant transformation, and altered gene expression patterns that lead to a reduction in cell proliferation and increased differentiation [12,13,14]. Some other breast cancer risk factors include lack of breastfeeding, early menarche, and late onset of menopause. Many studies have reported that modified risk factors such as obesity, alcohol consumption, and physical inability contribute to approximately 20% of global breast cancer incidence, and may offer the potential for reduction in the burden of disease through promoting a healthy lifestyle [15]. The rising issues after the treatment of cancer include sexual dysfunction, loss of strength, bone health, and physical and mental health [16,17,18]. Breast cancer is mostly epithelial in origin. However, it can be further subdivided into different subtypes depending on its biological behavior and its microscopic appearance, as displayed in Table 1.

Mammography is currently the most frequently used modality for the diagnosis of asymptomatic and at-risk breast cancer women at the age of 40. In conjugation with mammography, ultrasound is used for further assessment of suspicious lesions found and in patients with greater-density breast tissue. In addition, magnetic resonance imaging (MRI) is commonly used for high-risk patient screening [20]. On clinical breast cancer suspicion via breast mass palpation or abnormal mammogram, there is suitable investigative testing such as an image-guided biopsy, assessment of its staging, and appropriate therapy for targeting the malignant lesion [21]. Patients are classified into a subsequent Breast Imaging Reporting and Data System (BI-RADS) category that ranges from category 0 to 6, depending on the characteristics present on the mammogram, with 0 referring to an incomplete study that requires further imaging for evaluation, while category 6 represents biopsy-confirmed malignancy. Tomosynthesis, gamma imaging, and contrast-enhanced imaging are some emerging technologies that report increased cancer detection rates in conjunction with mammography. In these, tomosynthesis is the most likely candidate for future screening of breast cancer [22]. After malignant diagnosis, appropriate recommendations are made by surgical and medical oncologists, particularly breast malignancies specialists.

## 2. Methodology

This current review included the recent advances in the research of genomic instability in breast cancer to provide precise and comprehensive information on several aspects. All required information were in English and have been collected through electronic search of different sources including PubMed, ScienceDirect, SciELO, Google Scholar, and Web of Science. The selected articles’ reference lists were also checked to find relevant data. Titles and abstracts were screen independently to determine the eligibility of the study. Therefore, articles that were linked to incidence and recurrence of breast cancer, breast metastasis, mechanism of genomic instability in breast cancer, detection approaches, computational approaches, theranostic implications, DNA repair pathways, and PARP inhibitors were included. The study database encompassed peer-reviewed journal articles, books, theses, and review articles covering all aspects of genomic instability and breast cancer. Duplicate articles were identified with EndNote and excluded. WHO-, FDA- and CDC-related data were also included. 

## 3. Breast Cancer Metastasis

Breast cancer tends to spread to the bone, brain, liver, lungs, and distant lymph nodes, in addition to recurrence in the local area [23]. Seventy percent of patients with metastatic breast cancer had bone metastases, the most prevalent type of distant metastasis [24]. It is estimated that 30% of metastases are found in the liver, while the brain is the third most common site (10–30%) of metastasis [25]. Overall survival and organ metastasis tendencies for breast cancer subtypes are notably different. The Surveillance, Epidemiology, and End Results Program conducted a study on the association between breast cancer subtypes and distant metastatic sites [26] There is a strong correlation between bone metastasis risk and the HR+/HER2+ (luminalHER2) subtypes, according to these findings. The HR/HER2+ (HER2-enriched) subtype is more likely than the HR+/HER2 (luminal A and luminal B) subtype to metastasize to the brain. Additionally, the HER2-enriched subtype has a higher rate of liver metastases than the other HER2 subtypes. Patients with TN breast cancer are most frequently found to have pulmonary metastases. A multivariate analysis comparing different subtypes revealed that luminalHER2 and HER2-enriched subtypes had a much greater rate of brain, liver, and lung metastases than luminal A HER2-negative subtypes. Both basal-like and other TN subtypes are associated with a high risk of brain, lung, and distant lymph node metastases. The basal-like subtype, on the other hand, is associated with a low incidence of liver and bone metastases [27] Cancer cells accrue genetic mutations during tumor development and progression, which can result in the alteration of critical genes and pathways. A recent large-scale genomic evolution study of patients with breast cancer metastasis and local relapse revealed that metastases had a higher mutation burden than primary tumors, including inactivation of the SWItch/sucrose nonfermentable and Janus kinase 2-signal transducer and activator of transcription 3 (JAK2-STAT3) pathways [23]. According to another study, Notch pathway gene mutations/chromosomal inversions, fragile histidine triad gene mutations/rearrangements, and other shared changes in genes influence the immune response to metastatic cells [28]. As a result, organ-specific metastases from various primary cancer types may share genetic abnormalities to adapt to the same distant immune and host metabolic milieu. By comparing tumor cells in the primary site to distant lesions in the organ of interest in breast cancer animal models, tissue-specific gene signatures and signaling pathways have been found [29].

## 4. Genomic Instability and Its Consequences in Developing Breast Cancer 

Genomic instability is a hallmark of breast cancer because it develops several etiologies for this heterogeneous disease, and brings up an increased tendency to alter the genome [30]. Any alteration in several mechanisms such as cell cycle checkpoints, DNA repair machinery, mitotic checkpoints, or telomere maintenance results in genomic instability, as they are known to protect the integrity of the human genome [31]. 

Genomic instability is the most significant feature that empowers the functional competencies of cancerous cells for their survival, proliferation, and spread through several mechanisms at different times throughout tumorigenesis due to its ability to generate accidental transmutations and chromosomal reorganization. In some cases, functional loss of tumor-suppressor genes occurs as a result of accumulated errors induced by a deficient DNA repair system; while in other cases, it may lead to the stimulation of oncogene function and finally, promote cancer growth and development [32]. The proliferative capacity of tumor cells is sustained by various mechanisms, including the acquired ability of cancer cells to produce growth factors that can trigger self-proliferation in an autocrine manner, or by activating the surrounding normal stromal cell to produce growth factors in a paracrine manner [33]. Moreover, cancer cell proliferates by the downregulation of membrane-receptor-mediated signals, either by increasing the surface expression of membrane receptor or by the structural alteration of proteins that are responsible for ligand-independent activation of signaling pathways [32]. In breast cancer, a clear example of pathway inhibition is the human epidermal receptor (HER) pathway [34]. Human epidermal growth factor receptor 1 (EGFR/HER1), human epidermal growth factor receptor 2 (ErbB2/HER2), human epidermal growth factor receptor 3 (ErbB3/HER3), and human epidermal growth factor receptor 4 (ErbB4/HER4) are four members of the HER family, and the expressions of HER1 (30–40%), HER2 (20–30%), and HER3 (20%) are increased in the case of breast cancer instead of HER4 [35]. In breast cancer, the HER2 pathway is deregulated, and results in cellular functioning by several signaling pathways. The functional activation of the intracellular tyrosine kinase domain of HER2 occurs after the homo- or heterodimerization of the receptor, and leads to the activation of three major signaling cascades, Ras/Raf/MAPK, JAK/Stat, and PI3K/AKT/mTOR, which ultimately leads to cell proliferation, survival, division, apoptosis, migration, and metabolism [32].

The pathogenesis of breast cancer is associated with several molecular changes. Raised proliferative activity of a cell results in malignant transformation, and subsequently the accumulation of DNA replication errors accompanying an increased duplication rate of DNA [36]. Nevertheless, in response to several genotoxic events, the cell holds a mechanism to conserve the integrity of the genome. Under a genotoxic condition, the information from a DNA damage lesion is not transmitted by checkpoints to cell cycle regulators because the cell does not progress through the cell cycle [37]. Downregulation or mutation in genes such as ataxia-telangiectasia mutated (ATM), which are responsible for marking DNA damage as less severe, has been associated with the development of breast cancer [38,39]. The ATM protein belongs to a family of protein kinases containing three catalytic domains, ATM, ATR, and PI3K, also key components of the DNA damage response signaling cascade, which is encoded by the ATM gene to maintain the integrity of the genome inside the cell. To establish an association between DNA damage, progression of the cell cycle, and apoptosis, ATM primarily senses double-stranded DNA breaks and then phosphorylates and activates several proteins of downstream signaling pathways, including DNA damage repair, cell cycle arrest, and cell death [40]. In all nucleated cells, a normal physiological mechanism such as DNA repair is indispensable to maintain genomic integrity. A multifaceted, highly integrated, sensitive, and interconnected DNA damage response mechanism has developed, and appears to stimulate various cellular responses including DNA damage, cell cycle arrest, and cell death. The ATM protein, central to DDR, is a protein kinase that initiates the signaling pathway in response to double-stranded DNA damage [41]. ATM acts as a damaged sensor and a potential therapeutic target for cancer treatment. Additionally, ATM also participates in the normal processes in the cell such as metabolic regulation, transcriptional modulation, cell proliferation, oxidative stress, and degradation of proteins. ATM is expressed as nuclear serine/threonine protein kinase. In response to DNA damage, ATM is activated and phosphorylates several proteins, including p53, CHK1, CHK2, and BRCA1, which are important for DNA repair [42]. DNA damage activates the CHK1 protein which is the first line of defense against DNA damage. CHK1 arrests the cell cycle at the G1 phase by promptly modifying the CDC25 level and by altering kinase activity of cyclins at the S and G2 phases. The best-known modulator of CHK1 activity is a tumor suppressor named PTEN. Previous studies have also demonstrated that the PI-3K/AKT signaling pathway may regulate the phosphorylation of CHK1- and PTEN-deficient cells showing elevated phosphorylation of CHK1. In response to DNA damage, serine phosphorylation of CHK1 decreased the ability of the protein to be phosphorylated at another residue. As a consequence of lower CHK1 activity, PTEN-deficient cells are not able to regulate the level of CDC25, as well as cell cycle arrest [43]. Previous studies have reported that CHK1 is most commonly localized in nuclear compartments, while mutant CHK1 might be primarily seen in the cell cytoplasm. In addition, nuclear CHK1 could be seen departing from the nucleus toward the cytoplasm at the G2 phase of the cell cycle, and resulted in reduced CHK1 in the nucleus, which may elucidate the increased level of cdc25 at the G2 phase of the cell cycle. Further studies suggested that due to PTEN deficiency, reduced functional activity of CHK1 leads to a checkpoint defect at the G2 phase [44]. Lack of PTEN directly increases the kinase activity of AKT, which in turn stimulates CHK1 phosphorylation. After phosphorylation, CHK1 undergoes post-translational modification by the addition of the ubiquitin protein, which may inhibit CHK1 entry into the nucleus. In the cytoplasm, the sequestering checkpoint protein CHK1 spoils its normal function in DNA repair checkpoint initiation. Apart from checkpoint impairment at the G2 phase in PTEN-deficient cells, there will be an accumulation of DNA double-strand breaks due to the inactivation of the CHK1 [45]. 

Analysis of the checkpoint protein CHK1 in breast cancer cells specifies a raised level of CHK1 in the cytoplasm, along with deficiency of PTEN and increased phosphorylation of AKT. These observations propose that a lack of PTEN may induce defects in the normal functioning of the checkpoint protein, which triggers the signaling process of oncogenes. Decreased activity of CHK1 is highly associated with an increased risk of genomic instability and breast cancer. Additionally, AKT activation inhibits the key inhibitors, including p21, p27, and cdc25 of cyclins, which are responsible for arresting the cell cycle in response to checkpoint activation. Furthermore, AKT may induce MDM2 phosphorylation and activation to trigger the destruction of p53 [46,47]. Increased expression of CDC25A is the major substrate of checkpoint protein CHK1 has been seen in breast cancer. CDC25 overexpression may trigger the activation of the cyclin E/cdk2 complex, which may induce unscheduled replication origin firing, as well as initiation of chromosomal instability [48,49]. In response to the genotoxic-stress tumor suppressor, p53 stimulates various cellular DNA repair pathways responsible for preventing cell proliferation and inducing apoptosis [42]. Additionally, p53 is also responsible for transcriptional regulation of genes encoding different proteins involved in apoptosis and DNA repair. As a result of mutation or deletion, p53 declines its normal function and appears to play a critical role in the progression of breast cancer [50,51]. BRCA1 participates in the regulation of various genes’ expressions, which is associated with an increased risk of breast cancer. Cell-proliferation control, genome-integrity maintenance by recombination processes, and DNA repair are regulated by BRCA1 [52]. So, the mutation in BRCA1 also plays a causative role in the etiopathogenesis of breast cancer [53]. The genetic defect in genes that causes the response to DNA damage and DNA repair mechanism downregulation leads to genomic instability, which promotes breast cancer growth and development [54].

**Figure 1:**
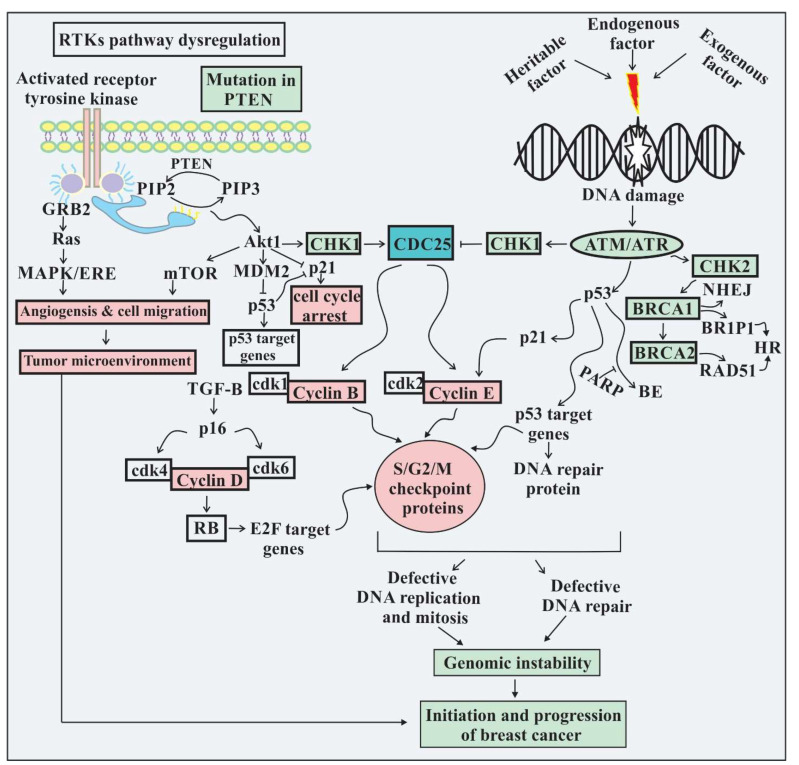
Schematic representation of genomic instability in breast cancer. In humans, inherited mutations in ATM, P53, CHK1, CHK2, and BRCA1/BRCA2 genes, which are DNA damage checkpoints, are associated with predisposition to malignancy. Primarily, the exact etiology of breast cancer is not clear, but various estimated factors, including heritable, endogenous, and exogenous, may cause DNA damage. Initiation of checkpoint response requires ATM or ATR kinase, depending upon the type of DNA damage. In response to DNA damage, ATM or ATR phosphorylate different substrates such as BRCA1, P53, CHK1, and CHK2. The caretaker tumor-suppressor genes BRCA1 and BRCA2 maintain the integrity of the genome by fixing errors mainly related to DNA double-strand breaks and replication forks. Breast cancer cells cannot repair the DNA strand breaks due to mutations in BRCA1 or BRCA2, which may result in accumulation of mutations that are responsible for triggering proliferation and metastasis. On the other hand, in response to DNA damage, BRCA1-mutated cells have a defective checkpoint function in the G1/S phase of the cell cycle, as BRCA1 also plays an important role in mediating cell cycle arrest. In DNA damage response, BRCA1 acting as a scaffold protein facilitates p53 phosphorylation by ATM, which leads to p53-induced p21 induction and mediates G1/S arrest. Primarily, this will result in defective DNA repair and chromosome instability, which further leads to acquiring genomic instability and malignant features. In breast cancer cells, germline or somatic mutation, copy number aberration, and epigenetic control alteration have been shown to affect several genes that are responsible for maintaining genomic stability in normal conditions. Loss of key DNA damage repair genes including BRCA1, BRCA2, RAD52, PALB2, BR1P; and genome caretaker genes such as ATM, CHEK2, and TP53 are highly associated with increased risk of breast cancer development. PI3-K/Akt, the extracellular signaling pathway, is crucial for the control of cell growth and is normally regulated by several extracellular signaling proteins, including insulin and insulin-like growth factors. In cancer cells, this pathway is abnormally activated by mutation in PTEN, and cancer cells can grow in the absence of such a signal, so this abnormal activation of Akt is central to the dysregulated growth process. The most common mutation of the tumor-suppressor gene in breast cancer is the loss of PTEN phosphatase, the normal function of which is to limit Akt activation by dephosphorylating PI3K. The tumor suppressor PTEN is an important modulator of chk1 function. Akt activation in response to PTEN loss phosphorylates CHK1 and leads to its monoubiquitination and sequestration from the nucleus. Outside the nucleus, phosphorylated CHK1 is unable to respond to its activating substrate, AKT/ATM, and there will be no phosphorylation of its substrate, including CDC25. CDC25A is the major substrate of chk1, which is overexpressed in breast cancer cells. In breast cancer, CDC25A overexpression leads to activation of the cyclin E/cdk complex, and has shown to play a very vital role in the unscheduled firing of origins of replication and induction of chromosome instability. So, reduced PTEN leads to increased Akt activation and increased cytoplasmic chk1 phosphorylation, thereby inhibiting its checkpoint function. A reduced amount of chk1 function is predisposed to genomic instability, and contributes to the development of breast cancer. In addition, Akt activation inhibits cyclin/CDK complex inhibitors, including p21, p27, and CDC25, which stimulate cell cycle arrest in response to activation of checkpoints. Moreover, activation of Akt phosphorylates and activating MDM2 stimulate the destruction of p53. In p53-deficient cells, the blockage of cdk2/cyclin E by p21 is not functional, thus hyperamplification of the centrosome may occur, which is the prerequisite for mitotic catastrophe.

Multiple changes in the genome such as amplification, deletion, and formation of translocation lead to heritable genomic instability in the cell or significant DNA damage, which ultimately results in malignancy [55]. The most lethal form of DNA damage is the double-strand break (DSB), which can be repaired by different repair pathways, including HR and NHEJ. DNA broken ends are joined by the NHEJ repair pathway without the identification of DNA sequence homology, and consequently, this pathway is extremely disposed to errors that occur during the cell cycle. The HR repair pathway is comparatively error-free because it depends on DNA sequence homology, and it prevails in the S and G2 phases of the cell cycle [56,57]. Covalently joined, inappropriate dicentric chromosomes may be caused by errors in the NHEJ repair pathway. During anaphase, these dicentric chromosomes may break, and through further NHEJ repair, leads to the formation of a new dicentric chromosome [58]. This whole process of dicentric chromosome formation is called the breakage fusion bridge cycle (BFB) and plays a critical role in causing genomic instability related to telomeres [59] (Figure 1). In many cancers, especially breast cancer, BFB may be responsible for causing genomic instability, because BFB cycles are self-propagating [60]. The HR repair pathway includes products of numerous genes, many of which are involved in stalled replication fork repair [57]. The tumor suppressors BRCA1 and BRCA2 play very vital roles in maintaining genomic stability, as they are the key players in the HR repair pathway. Linkage studies in families with breast cancer at an early age suggest that mutation in BRCA1 and BRCA2 is linked to breast cancer predisposition [60]. In one meta-analysis, breast cancer risk for mutation in BRCA1 and BRCA2 was 57% and 49% respectively [61]. Additionally, the hereditary mutation in BRCA1 and BRCA2 is associated with prostate, pancreatic, and colon cancer [62]. Moreover, BRCA1 and BRCA2 germline mutations are linked to different breast cancer subtypes; for example, cancer associated with BRCA1 is of the more aggressive triple-negative breast cancer subtype, whereas BRCA2-associated cancer is mostly related to the hormone-receptor-positive subtype of breast cancer [60]. 

## 5. Theranostic Interpolation Approach of Genomic Instability in Breast Cancer

At present, genomic instability can be detected through many diverse technologies that can range from single-cell approaches to high-throughput multicellular techniques. These technologies all have the capability to detect different levels of changes in the genome. However, no assay can measure the small chromosomal change rate, including deletions, amplifications, and within-cell population in any version. So, there is a prerequisite for the need for sensitive, high-resolution technologies that can detect genomic instability over time. Finding more effective therapeutic solutions via capitalizing on differences between cancer and noncancer cells is an ongoing and intense area of research.

## 6. Detection of Genomic Instabilities in Breast Cancer

The genomic instability in cancer can be detected via numerous strategies. It should be noted that genomic inability refers to the rate of chromosomal changes, and is therefore a measure of variability in the state of chromosomes between each cell within the tumor. Repeated cell-population measurements throughout the tumor evolution are required for accurate instability assessment. Preferably, individual cancer cell measurements are necessary to define the exact rate of genomic alteration or variability for a specific cancer. However, this measurement is more easily obtained for cancer cell lines, but it is more difficult to measure accurate genomic instability in clinical specimens of the tumor; as they have limited material, along with substantial cellular heterogeneity. Few studies have characterized true genomic instability via determining the accurate chromosomal alteration rate in various cancer types [63]. Due to the difficulty of measuring actual genomic instability, numerous methods have been used as a surrogate to estimate the extent and frequency of genomic alteration for static cancer cell populations for describing the genomic instability. Therefore, while claiming to interpret the genomic instability in cancer, precautions must be taken. As we know, genomic instability is linked to any genetic level, so the methods that can detect any change in chromosomes, microsatellites, or nucleotides are adequate for the assessment of genomic instability components. Such methods for detection includes karyotyping, flow cytometry, single-nucleotide polymorphism (SNP) arrays, genome sequencing, and polymerase chain reaction (PCR) (Table 2).

The techniques that can analyze growing cultures triggered from a single cell at a regular time; i.e., serial sample analyses, are used to assess chromosomal instability, while single-cell tracking is utilized for monitoring multiple cells and their progeny simultaneously within an experimental population. For assessing the chromosomal instability rate prerequisite, techniques that have no adverse effect on cell viability or proliferation are needed; therefore, live-cell imaging is employed. The chromosomal visualization can be achieved in numerous ways, including fluorescent labeling of chromatin-associated proteins, fluorescently labeled artificial chromosomes, fluorescent operator/reporter systems, and modified gene-editing systems. 

## 7. Fluorescent Labeling of Chromatin-Associated Proteins

Genetically encoded fluorescent tags are used for chromosome labeling for live-cell imaging for detecting histones and other chromatin-related proteins. This technique is unable to measure chromosomal instability per se, as it does not allow the tracking of any gain or loss in a particular chromosome, as it uniformly labels all chromosomes. This approach allows the evaluation of the dynamics of the chromosome as the cell progresses through the mitotic process, which unwraps aberrant mitotic changes that include all phenotypic-associated chromosomal instabilities, chromosomal congression or segregation errors, anaphase bridges, breakage of chromosomes, and decompaction [64]. Though this technique conventionally relies on the manual inspection of an image, which can be lengthy and time taking, software-driven automation and analyses are possible via single-cell tracking tools included in many image-capture software packages [65]. 

## 8. Fluorescent Reporter/Operator System

This approach enables numerical CIN quantification within the live cells. They contain a fluorescently tagged DNA binding protein, the reporter, that binds to a stably integrated DNA element, called the operator, within the genome at a distinct locus of the chromosome. The number of fluorescent foci present within the cell can be used as a surrogate marker for the chromosome copy number, and this can be monitored via multiple cycles of cell division. Therefore, to evaluate N-CIN, both population heterogeneity and the temporal dynamics of copy-number losses or gains can be quantified. This technique does not detect events involving nonlabeled chromosomes, and is also incapable of assessing structural CIN [66]. 

## 9. Human and Mouse Artificial Chromosomes

Rather than introducing a transgenic marker into an endogenous chromosomal locus, a related approach involves the use of human or mouse artificial chromosomes engineered to contain informative reporting genes in order to enable the assessment of HAC or MAC copy-number changes through flow cytometry or QuantIM. These artificial chromosomes include centromeric sequences that form functional kinetochores, and they rely on the same segregation machinery as endogenous chromosomes [67]. So, the increased rate of change in human artificial chromosome copy number or mouse artificial chromosome copy number indicates an enhanced whole chromosome mis-segregation or N-CIN rate. However, these systems allow the evaluation of either gain or loss of HAC or MAC theoretically, but until now, they practically are only designed for the assessment of chromosomal loss [68]. The fundamental limitation of these approaches is that they cannot detect changes involving endogenous chromosomes directly, so they are not able to differentiate the loss or gain rate of a particular chromosome. These techniques assume a consistent mis-segregation rate for all endogenous chromosomes that are equal to the HAC or MAC mis-segregation rate. Interestingly, mouse artificial chromosomes (MACs) are maintained more stably in some cell types, showing that human artificial chromosomes (HACs) may have inherent instability levels in certain contexts. These approaches are suitable for research-based applications and the most effective preliminary screening tools [69]. 

## 10. Modified Gene-Editing Systems

To visualize some particular loci, many emerging gene-editing technologies are employed, including zinc-finger nucleases (ZFNs), CRISPR/Cas9 systems, and transcription activator-like effector nucleases (TALENs) [70]. Generally, for standard gene-editing purposes, these methods contain an endonuclease that is directed to a specific locus through a target-recognition sequence. In zinc-finger nuclease and transcription activator-like effector nuclease, the target recognition and endonuclease activity are provided by a single protein, while CRISPR typically employs the Cas9 endonuclease and RNAs for targeting a gene [71]. 

## 11. Single-Cell Genomic Approaches

Recent advances in scientific research have made it possible to conduct genomic analyses at a single-cell level, permitting the evaluation of both numerical and structural chromosomal instability at an unprecedented resolution. These are called single-cell CGH, single-cell whole-genome sequencing (sc-WGS), single-cell whole-exome sequencing (sc-WES), and single-cell copy number variation (sc-CNV) analyses. Presently, most single-cell genomic approaches require isolated single cells and their amplified DNA before any further analyses. Not all genomic regions being identically amplified is the common limitation to these approaches. Various single-cell isolations and techniques for DNA amplification are available at present. These approaches have continued to evolve toward an improvement in the capture rate of tumor cells, as well as an enhancement of the amplification of DNA [72,73]. Moreover, innovative single-cell whole-genome techniques have emerged that bypass the requirement of DNA preamplification [74]. Gonzalez-Pena and Natarajan et al. [75] investigated a new single-cell WGA using primary template-mediated amplification (PTA), and showed greater coverage of genome coverage and variant detection from single cells.

## 12. Multicellular Approaches

The cytogenetic approaches, usually used for the detection of structural chromosomal instability and N-CIN, are extensively employed in clinical and research areas. These approaches include karyotyping analysis, fluorescence in situ hybridization (FISH), spectral karyotyping, and multiplex-banding techniques. In addition to these, quantitative and high-throughput imaging cytometry is also used to quantify singular cell data from a huge number of cells, as it increases the experimental throughput. The two commonly used quantitative high-throughput cytometry approaches are quantitative imaging microscopy and imaging flow cytometry [76]. Another multicellular approach to detect microsatellite instability is known as polymorphism chain reaction (PCR), which amplifies the specific region, thereby comparing the lengths of tumor DNA short tandem repeats and normal DNA to assess the MSI state [77]. 

## 13. Computational Identification of Mutations Leading to Breast Cancer

Cancer can initiate, progress, and metastasize due to mutations, particularly genomic insertions or deletions, single-nucleotide variants (SNVs), structural variants, and epigenetic changes [78]. The advent of technologies has led to the progress of whole-genome sequencing and genome analysis of tumor cells in collaboration with the International Cancer Genome Consortium (ICGC) and The Cancer Genome Atlas (TCGA) [79,80,81,82]. These projects have successfully identified and reported numerous somatic SNVs responsible for various cancer types, and especially, they have developed a comprehensive landscape representing all reported somatic mutations in breast cancer patients [82]. The identified set of mutated genes and their associated pathways among the most prominent cancer types have initiated the functional assessment of tumor cells, though this area still needs to realize a lot of progress, and initial development cannot be ignored either. The main challenge is to determine the underlying mechanisms differentiating driver mutations from passenger mutations. Mutations that selectively progress the growth of tumor cells are termed driver mutations, whereas inert mutations, which usually do not deliberately participate in tumor cell growth, are called passenger mutations [83]. 

The functional impact of SNVs is mostly studied for coding regions, and the characterization of such changes is usually predicted by employing various computational techniques regarding how a mutation will alter the normal phenotype of the cell or molecule performing a critical role in regulatory pathways. However, a major number of mutations reported within cancer samples and tumor analysis resides within noncoding regions [84]. Such noncoding mutations directly affect the transcription factor binding, as well as the activity of RNA-binding proteins and even miRNAs, which in return affect the gene regulatory mechanism, transcription, methylation, protein stability, and translation [85]. Mutation analysis and prediction of functional SNVs require computational pipelines to speed up the analysis; while they require experimental validation and characterization of SNVs and their role in cancer progression, such integration of computational and experimental techniques has proved to be a good analysis technique in various studies [86,87,88]. Generally, functional SNVs are predicted via a common pipeline that includes the identification of somatic SNVs, analysis of regulatory regions, comparison of SNVs with common germline variants, and prediction of altered transcription-factor binding sites. Somatic SNVs are identified by comparing tumor genome sequences with normal tissues. Usually, this is quite challenging due to the low frequency of somatic SNVs, and they should be distinguished from common sequencing errors [89]. Thus, this overall requires high-end sequencing techniques, which may increase the cost as well. Some commonly used tools for the identification of somatic SNVs include GATK [90,91], which identifies tumor-specific SNVs by comparing normal and tumor samples; and MuTect [90,92], which uses statistical or heuristic models to analyze tumor samples. These algorithms usually employ single methods to identify SNVs, whereas some approaches use a combination of methods to increase efficiency as well [93]. 

Among millions of identified SNVs, only a few are diverse, and to detect such functional mutations currently, two strategies have been implemented. One is to target the regulatory DNA elements, which may include promoters, transcription initiation sites, histones marks, etc. [89]. Some studies only focused on cancer-related regulatory elements, because restricting the overall analysis to only targeted regulatory elements empowers the detection technique by reducing the search space for SNVs. Another strategy in use to identify the SNV clusters instead of single mutations within small DNA fragments is called hotspots. The advantage of the identification of such mutational hotspots within small DNA windows is to increase the frequency of marking and reduce the dimensionality by comparing the SNV frequency of the window with the whole distribution of SNVs. This technique is adopted either locally or globally; i.e., the comparison of SNV frequency either to the close genomic regions or to functionally similar regions. The DNA window size may also vary depending on the nature of the computational power, and may range from 50 to 500 bp. Though small windows may provide a higher resolution than longer ones, which are usually associated with gene expression levels or replication timings [94]. To increase the efficiency of the detection technique, both strategies are often used in combination. 

Moreover, there are special centers and programs designed to facilitate cancer research and to develop a better understanding of the disease and responsible factors, as well as to predict novel therapeutic options. The German Consortium for Hereditary Breast and Ovarian Cancer (GC-HBOC) is a center that specializes in genetic screening, counseling, and providing healthcare benefits to breast and ovarian cancer patients [95]. These centers are facing the major challenge of classifying the uncertain significance of variants in various cancers and maintaining a central patient registry to define all the genetic aberrations. In addition to that, breast cancer patients are screened for BRCA1/2 mutations, which may determine the severity of the disease and determine the therapeutic options and response time of the patient. To analyze the mutations crucially and to avoid any chance of missing information, automized predictions employing in silico approaches, machine learning, and artificial intelligence have become frequently adopted techniques. Existing approaches classify the mutations based on the difference between biochemical properties of replaced amino acids and the location of mutations among highly conserved regions. For such types of analyses, computational tools adopt structure- or sequence-based techniques, which may rely on assumptions as well [96,97,98]. 

Here, we will briefly discuss four commonly used prediction tools of medical genetics, including SIFT [99], Align-GVGD [100,101], MutationTaster2 [102], and PolyPhen-2 [103]. Align-GVGD takes input in the form of aligned sequences. It then computes scores for each aligned column indicating a substitution. For each column, a biochemical distance score is calculated based on the extension of the pairwise Grantham difference (GD) and conservation score following Grantham variation (GV). Seven different classes of substitutions are then formed based on these scores, which indicate the mutation having the least likely to most likely interference with function [100,101]. SIFT, based on sequence analysis, determines evolutionary conserved amino acids within a protein, and classifies nonsynonymous single-nucleotide polymorphisms (nsSNPs) on their bases. It calculates the probability for each amino acid substitution to occur at each position of the aligned sequences, and threshold values are assigned. Amino acids having scores less than the threshold value of 0.05 are predicted to be missense variants that can damage the protein both structurally or functionally [99]. MutationTaster2 classifies the variants into either polymorphisms or disease-causing, based on regulatory features, splice sites, and evolutionary conservation. It also connects with different databases to classify mutations as polymorphisms or disease-causing [102]. The Polymorphism Phenotyping v2 (PolyPhen-2) tool for the naive Bayes classifier is also based on sequence analysis, but can also analyze structural features if the 3D structure is known [103]. 

For the past several years, various authors have published comparisons of available computational tools and in silico prediction methods, and studied their functional impact to provide a comprehensive picture of these applications [104,105,106,107]. Though each tool has its strengths and weaknesses, but the latter can be overcome by combining the results of various approaches and predicting a concise result on its basis. 

## 14. Pathway Analysis of Tumor Cells Leading to Genomic Instability

Genome analysis alone has several gaps in the core identification of protein function; thus, research has been focused on the regulation of signaling pathways in cancer cells. It has also become a vital component in the prediction and advancement of targeted therapies. Numerous studies have been reported to unravel the underlying signaling cascades in breast cancer. A study reported a pathway analysis of a genome-wide association study (GWAS) especially in breast cancer, and highlighted pathways highly associated with genetic alterations of breast cancer. These pathways included signaling of hepatocyte growth factor receptor, growth-hormone signaling, and syndecan-1-mediated signaling. Genes from these pathways were then checked for their associated SNPs, and their enrichment score represented the gene-based associations. Finally, overlapping genes were marked using hierarchical clustering, and the adaptive rank-truncated product (ARTP) method was adapted to identify clusters having more associative signals. Thus, pathways having regulatory genes with the most effective roles in breast cancer could be identified. Similarly, the authors reported the RAS/RAF/MAPK canonical signaling cascade to be highly associated with breast cancer susceptibility [108]. 

Pathway analysis and protein–protein interactions (PPIs) have also been studied to identify the key genes of breast cancer. Wang et al. introduced a method called BridGE to classify structured motifs within genetic networks, especially those derived from GWAS studies. Reverse-genetics studies have revealed that the members of a particular pathway share patterns that can be searched to predict pathway interactions within a population. The bridGe is based on this principle, and it has been applied to a total of six breast cancer cohorts to identify significant pathways and their interactive members. They overall predicted vitD receptor, mitotic prometaphase, purine metabolism, a steroid hormone, and glutathione conjugation as the major biosynthetic pathways related to breast cancer risk [109].

Triple-negative breast cancer (TNBC) patients cannot express the progesterone, estrogen, and HER2 receptors, and have a poor prognosis history; thus, researchers have also been focused on discovering the underlying mechanisms of TNBC. Pathway analysis has potentially helped to identify the initiation and developmental process of TNBC by comparing expression profiles of genes of TNBC patients with non-TNBC ones. These genes were then analyzed via gene ontology and the Kyoto Encyclopedia of Genes and Genomes (KEGG) pathway, and their protein interactions were then modulated. The authors calculated the correlation between survival rate and gene expression levels to discover potent targets for diagnosis and prognosis, which later on could also be evaluated as potential therapeutic targets as well [110]. 

Moreover, transcriptome data analysis also lays a good foundation to discover key regulatory elements in a particular pathway or network. Numerous studies have been designed to analyze transcriptomic data to decipher complex signaling networks involved in various diseases, especially cancers [111,112,113]. RNA-seq transcriptome analysis of BC patients from the Gulf region by integrating NGS data analysis with pathway analysis to highlight some important functional and signaling perturbations during BC development [113]. They observed some common signatures of BC-derived genes among the transcriptome of BC patients from the Gulf region, as well as differentially expressed genes from the TCGA BC dataset. 

## 15. Computational Prognostic Indicators for Breast Cancer

Breast cancer prognosis has always been a challenge due to its complexity and a lack of uniform models, as well as the incomplete analysis of datasets. Computational models based on biological implications can contribute significantly by integrating significant information on chromatin modification, transcriptomic alterations, and stroma response, which in return may help to determine the prognosis of breast cancer patients, and will provide valuable insights into the oncological determinants [114]. The lack of precision molecular indicators is the main reason to impede the personalized and specified therapeutic interventions. An ideal (sensitive, specific, and cost-friendly) prognostic indicator can be designed by employing integrated computational models based on clinical variables, which can significantly increase the understanding of heterogeneity and the complex biology of breast cancers. Moreover, prognostic indicators are expected to clearly define the risk groups associated with individuals in terms of tumor progression, metastasis, invasion, or response to a particular treatment [115]. 

The most common prognostic indicators of breast cancer are BRCA1 mutations, especially for early diagnosis, and they serve as a predictive biomarker for almost 80% of patients with combined ovarian and breast cancer and up to 20% of women with a family history of breast cancer [116]. Enhanced expression of cohesion and RAD21 is another prognostic factor associated with increased resistance to chemotherapy, mostly in HER2, basal, and luminal breast cancers [117]. Additionally, expressions of anterior gradient-2 (AGR2) protein and muscle segment homeobox 2 (Msx2) are also observed for prognosis in breast cancer patients [118,119]. 

The Cox proportional hazards regression model is another commonly used computational approach to predict critical biomarkers [120,121]. It calculates a survival function to produce the probability of death or survival given at a time after diagnosis. Some other computational methods have also been proposed in the same context, including Bayesian network analysis and support vector machine methodologies. Bayesian network analysis of signaling pathways facilitates the evaluation of a probabilistic relationship among candidate genes within the network [122]

Moreover, transcriptomic microarray and sequence data analysis provide multigene signatures [123], which not only contribute to prognosis, but also can predict tumor sub types and their specific resistance to chemo or radiation [124]. Such multigene models generally classify patients by indicating the particular outcome of the disease or treatment response according to the disease pathology; e.g., the DNA content of patients with breast cancer classifies them in either stable or unstable (conferring a good or poor prognosis) states [125]. In addition, machine-learning algorithms have been successfully applied to identify multigene signatures and pathways associated with breast cancer. These algorithms are usually applied to mass spectrometry profiles or transcripts, and are usually characterized in three main groups, which include: (1) supervised classifiers; (2) unsupervised data mining; and (3) semisupervised models. Supervised models (for example, prediction analysis of microarray (PAM) or decision-tree [126] models are trained for sample labels, including good or poor outcomes, before making a decision. Results of semisupervised models are based on input labels and raw data, which allow partial labeling. The choice of model selection truly depends on the type of data provided for the analysis. Some computational methods based on gene expression to identify prognostic determinants in breast cancer are given in Table 3. 

## 16. Computational Approaches to Facilitate Precision Medicines

Precision medicine can be the root of the cancer treatment if fully guided by the tumor’s genomic alterations. Its hypothesis has sparked major goals in oncology, and has led to the expedited initiation of whole-genome sequencing, genome analyses, development of cancer databases, and new tools for statistical analysis of data. Such an advent of computational technology has embraced much appreciation in the field of science and technology, which led to research focused on altered genomes and targeted therapies [139]. This eventually will lead to the delivery of the right drug to the right person [140]. Precision oncology is not only based on the genome sequence of an individual; rather, it also targets blood typing, target proteins, inhibitors, biomarkers, and pathway analysis [141]. Even with the high advancements in sequencing technologies and computational frameworks, precision medicine still lacks a defined outcome in clinical trials, as it has been reported that only 10% of mutations are actionable [142]. Moreover, tumors also depict remarkable diversity in their genomic alterations, and thus provide a heterogenous mutational landscape as a challenge. 

Precision medicine is one of the greatest challenges in the field of pharmacogenomics, which aims to produce effective drugs based on specific genetic signatures. Driver mutations identified via next-generation sequencing, along with the molecular targets, forecast how a person will respond to a particular treatment or drug. One common example of precision pharmacology is trastuzumab (Herceptin), a breast cancer drug that targets patients with overexpressed human epidermal growth factor receptor 2 (HER2) [143]. Precision pharmacology aims to screen compounds with the aid of genomic information to target a specific group of genetic profiles, rather than having broad action against many diseases. This includes genomewide sequencing; statistical analysis, which correlates the genomic data with clinical observations; and identification of actionable driver mutations. This process is halted or delayed because of the absence of databases and the availability of specific data facilitating the understanding the diagnosis and prognosis in cancer biology. 

Various tools and computational pipelines have been designed to analyze massive clinical, genomic, and proteomic data [144,145]. Statistical correlation can help to deal with massive experimental observations, whereas bootstrapping and cross-validation techniques can help to predict the key variables. Coupling computational, theoretical, and experimental biology can unravel the major mechanisms responsible for genome alteration, interactions of proteins, DNA damage, and drug development. Merging all this data will articulate the path toward the development of targeted medicine, and may prosper in treating cancers with greater accuracy and precision. 

## 17. Therapies Targeting DNA Repair Pathways

Currently, few chemotherapy drugs have been reported that can be used as direct therapeutic agents in DNA repair. DNA repair inhibitors cause the fragile DNA repair system to break down, which leads to the demolition of cellular homeostasis, which in turn results in cancer cell death [146]. One of the DNA repair inhibitors is the O6-methylguanine-DNA methyltransferase (MGMT) inhibitor. MGMT is a DNA repair protein that detaches the alkyl group, and is an early target in developing a DNA repair inhibitor [147]. 

## 18. PARP Inhibitors

PARP inhibitors are emerging chemotherapeutics that show significant activities in various cancer treatments, including breast cancer. The PARP substrate is the main target of their inhibitors, with a goal of hindering the DNA repair pathway in the cancer cells. The first-ever PARP inhibitor that received monotherapeutic approval was Olaparib (AZD-2281) for treating ovarian cancer in females with a *BRCA* mutation. Homologous recombination-mediated DNA repair is upregulated via PARP inhibitors, as they induce RAD51 foci formation [148,149]. This explains why PARP inhibitors may be synthetically lethal to nonfunctional homologous recombination cells. Any tumor having a defect in the homologous recombination function would show synthetic lethality with PARP, which subsequently displays several gene mutations, including *BRAC1, BRAC2, ATM, RAD51, RAD54, XRCC2, XRCC3, DSS1, RPA1, CHK1, CHK2, ATR, FANCA, MRE11,* etc. [150]. Moreover, epigenetic silencing of BRCA1 function induces hypersensitivity to PARP inhibitors. The PARP and homologous recombination synthetic lethality may be extended to nonhomologous recombination components that modulate their protein function. For example, PTEN mutation is sensitive to PARP inhibitors due to RAD51 modulating expression and cell cycle checkpoint regulation [151,152]. Other DNA repair genes that are not associated with homologous recombination function, shown defective as the result of PARP inhibitor sensitivity, are XAB2, CDK5, CDK1, DDB1, PLK3, MAPK12, PNKP, Lig1, STK36, and STK22c [153,154,155]. Most PARP inhibitors predominantly trap PARP on DNA-sensitizing alkylating agents, while some show their activity in inhibiting the catalytic activity of PARP via topoisomerase I inhibitors [156]. 

The immune microenvironment of DNA-repair-deficient tumors is generally immunosuppressive, with exhausted T-cell infiltrate expressing high levels of checkpoints. The targeted cell death caused by PARP inhibitors has the potential to reset the tumor microenvironment and polarize the immune response toward a T_h1_ antitumorigenic profile, resulting in a shift from immune escape to the elimination of the tumor [157]. Therefore, PARP inhibitors represent a promising combination therapy with therapies targeting immune checkpoints. The development of PARP inhibitors has led to a surge in interest in other DNA repair protein inhibitors as monotherapy or sensitizing agents. They include inhibitors of poly(ADP-ribose) glycohydrolase (PARG), which is involved in the process of catalytic activity of PARP reversal before DNA repair completion. Breast cancer cells deficient in *BRCA2* were reported to be sensitized to PARG inhibition [158,159]. Other DNA repair protein inhibitors include APE1 inhibitors, ATM and ATR inhibitors, RecQ helicase inhibitors, DNA-PK inhibitors, and FEN1 inhibitors [160,161,162,163,164,165]. However, the changes in these DNA repair proteins that are therapeutically relevant to cancer are not obvious. PARPi is generally well tolerated, but Chk1 inhibitors are associated with serious adverse effects and limited biospecificity (Table 4). The ATM/ATR inhibitors are in trial stages. It is also a challenge to recognize DNA damage response proteins that bear selective toxicity to cancer cells. Mutations or alterations in expression do not result in a changed function. To deliver the utmost therapeutic advantage from DNA damage-response therapies, the development of functionally applicable biomarkers is crucial to delivering accurately précised medication. 

## 19. DNA Repair Pathway

DNA damage, if left unchecked, may predispose one to malignant transformation of the cell. Multiple pathways function in cells to correct such errors by either initiating DNA repair or apoptosis. DNA repair pathways were found to be recruited within the cell immediately after DNA damage. They can be categorized as the nuclear excision repair pathway (NER), base excision repair pathway (BER), mismatch repair pathway (MMR), and DNA double-strand-break repair pathway (DSBR) [166].

The nucleotide excision repair pathway (NER), consists of 25 proteins that are activated to replace modified nucleotides in damaged DNA with the correct ones. DNA damage caused by exogenous chemicals, UV light, and chemotherapeutic agents is repaired by this pathway. NER operates through the following steps to repair DNA. Firstly, there is recognition of the damaged site. Secondly, incision of involved strand takes place. Thirdly, DNA is synthesized; and lastly, there is ligation of disconnected wings by the DNA ligase enzyme [167]. Three distinct pathways operate under the NER pathway. These are the transcription-coupled repair pathway (TCR), global genomic repair pathway (GGR), and differentiation-associated repair pathway (DAR) [168].

In the base excision repair pathway (BER), there is a substitution of modified bases with correct ones by the processes of methylation, deamination, and oxidation [169]. A specific DNA glycosylase enzyme that can recognize and excise modified bases from the genome removes these structurally changed bases from the DNA. New bases are synthesized by polymerase delta or epsilon, and the remaining cut ends are ligated by the DNA ligase enzyme [170]. Many SNPs are involved in the core base excision repair protein and DNA glycosylase genes in different malignancies, such as breast cancer. The BER efficiency is found to be a chief determinant of BC risk, because it works in oxidative DNA damage repair that is induced by free radical production during cellular estrogen metabolism or exogenous exposure. Numerous studies have reported the relationship of polymorphism with BER genes (XRCC1, APEX1, and OGG1) and breast cancer [171,172]. 

The mismatch repair pathway (MMR) is responsible for two types of mismatches in the cell. First, the base mismatches’ excision is caused by endogenous and exogenous agents, responsible for base methylation, deamination, and oxidation. Second, it also corrects base-to-base mismatches originating from replication and insertion or deletion errors [173]. This system functions by recognition of lesions, strand distinction, excision, and then repair [174]. MMR gene variants may predispose one to breast cancer. SNPs play a pivotal role in genomic integrity, so SNPs in MMR genes contribute to susceptibility to breast cancer risk. Genetic aberration in the prime MMR genes hMLH1 and hMLH2 were found to be associated with sporadic breast cancer [175].

A double-strand-break repair system (DSBR) repairs broken DNA strands. This pathway is further divided into nonhomologous end-joining repair (NHEJ) and homologous repair (HR). In the NHEJ repair process, broken ends of DNA strands are ligated by a specific ligase enzyme. This pathway is deprived of a homologous sequence control system. As a consequence, there is a possibility of errors, such as inversion or deletion [176]. HR functions in error-free mode as broken ends are repaired according to the homologous DNA sequence [177]. The selection of pathways for repair is determined by whether RAD 51 or KU binds to the damaged site. In the case of binding of RAD 51, the NHEJ pathway is activated, and if KU binds, the HR pathway is activated. Many proteins involved in homologous recombination are dysregulated in the case of breast cancer. RAD 51 is overexpressed or deregulated in invasive ductal breast cancer, triple-negative breast cancer, and bilateral breast cancer. Variation in the Rad21 gene is probably involved in hereditary breast cancer development. The precise role of BRCA1 in homologous recombination involves 5′ to 3′ DSB resection to form 3′ ssDNA overhangs and load RAD51 to ssDNA [178]. Many studies have highlighted the genotypic polymorphisms of genes participating in nonhomologous recombination, including KU70, KU80, XRCC4, DNA-PKcs, and their linkage with an increased risk of breast cancer [178].

## 20. Conclusions 

Breast cancer is an alarming heterogeneous disease; substantial by the profile of gene expression, modified genomic patterns, along with fluctuated molecular markers, leads to epigenetic aberrations that alter the growth and survival pathways. The genomic profile of breast cancer is essential for the prognosis of the disease due to the association between DNA mutation, genomic instability, and nucleotide modification. Many researches on cancer are focused on finding a definite cure. An integrative computational model of omics data and clinical variables may significantly improve the understanding of the biology and heterogeneity of breast cancer. It also could help in identifying cancer-related genes and pathways for cancer initiation and development. Further efforts through various approaches toward the breast cancer genome, amplifiers, and complex patterns could identify selective vulnerabilities created by underlying instabilities, which may not only enlighten the precise genomic instability mechanisms and reasons, but also lead to new treatment regimens and methods regarding the definite cure of breast cancer. 

## 21. Limitations and Future Perspective

Despite of extensive research on breast cancer genomic integrity and genomic instability, the ubiquitous mechanism for the initiation of tumors still remains a mystery. All hypotheses probably could be true in different contexts. Research into the prospect of activating checkpoints that monitor genetic integrity has made great progress. Continued sequence-level research of amplicon architecture may provide insight into the amplifier pattern’s underlying cause. Additionally, it is unknown whether amplification is primarily a temporary activity (occurring during telomere crisis) or a continuous process altering tumor genomes. Future research should elucidate the nature of a potential failure in DSB repair in complex-pattern breast cancers, as well as the functioning of BRCA1 and BRCA1 pathway components. Future investigations should target selective vulnerabilities induced by underlying genetic instabilities in both amplifiers and complex-pattern cancers. It is also known that the cancer-associated patterns of genetic alterations are complicated and multilayered, which makes it difficult to personalize therapy decisions in specific instances. Understanding these nuanced patterns of interindividual heterogeneity is critical to developing innovative medications, predicting therapy responses, and identifying diagnostic and companion biomarkers for better breast cancer care.

## Figures and Tables

**Table 1:** Breast cancer types [19].

Breast Cancer Type	Incidence	Features	Prognosis
Infiltrating ductal carcinoma	70%−80%	⮚ Presence of ductal carcinoma in situ Characteristics of a cell vary Solid tumor The appearance is speculated and irregular	⮚ Grade- and stage-dependent
Infiltrating lobular carcinoma	15%	⮚ Solid tumor in texture The appearance of cells in a single pattern order Estrogen receptor (positive) while human epidermal receptor 2 (negative)	⮚ Similar prognosis to IDC Metastasis differs from IDC
Tubular carcinoma	1–5%	⮚ Estrogen receptor (negative) while human epidermal receptor 2 (positive) Small and tubelike structure formation Not palpable	⮚ Better prognosis than infiltrating ductal carcinoma Rarely lymph node metastasis
Invasive papillary carcinoma	Less than 1%	⮚ Soft tumor texture Have fingerlike projections	⮚ Prevalent in postmenopausal women Good prognosis
Colloidal carcinoma	Less than 1%	⮚ Nonpalpable tumor Cells surrounded by excess mucin Soft tumor texture	⮚ Lymph node involvement Low occurrence in young age Good prognosis
Medullary carcinoma	Less than 1%	⮚ Soft tumor texture Sheetlike cells Triple-negative tumors	⮚ Frequent in young women BRCA1 mutation carriers

**Table 2:** Different methods of detection of genomic instabilities.

Detection Method	Cellularity	Detected Alterations
Karyotyping	Single-cell	Complete and segmental chromosomal instability, aneuploidy
Single-cell sequencing	Single-cell	Complete and segmental chromosomal instability, translocations, insertions, deletions, and mutations
Flow cytometry	Multicell	Cell ploidy/aneuploidy
Comparative genomic hybridization array	Multicell	Complete and segmental chromosomal instability
SNP arrays	Multicell	Complete and segmental chromosomal instability, single-nucleotide polymorphisms, uniparental disomy, loss of heterozygosity
PCR	Multicell	MSI, mitochondrial instability
Whole-genome sequencing	Multicell	Complete and segmental chromosomal instability, translocations, insertions, deletions, and mutations.

**Table 3:** List of methods for identification of prognostic determinants in breast cancer.

Data Source	Methods
Gene expressions (based on the experiment)	Expression levels Hierarchical clustering “Leave-one-out” cross-validation [127]
Gene expressions (based on text mining)	eScience–Bayesian [128]
Gene expressions (based on clinical and genetic markers)	I-RELIEF [129] (Iterative method based on the feature selection algorithm)
Gene expressions (based on copy number and pathway)	iCluster [130] PARADIGM [131]
Genomewide gene expression	RXA-GSP [132] LDS [133] MSS [134] BCRSVM [135] Correlation [136] PAM [137] Cox proportional-hazards regression modeling [126] Bayesian network analysis [138]

**Table 4:** Major PARPi for the treatment of breast cancer.

PARPi	FDA Approval	Catalytic Sites	Patient Population
Olaparib/AZD-2281	Approved	PARP 1,2,3	HER2 negative, homologous recombination deficiency, TNBC and/or germline BRCA mutation, stage I to III
Niraparib/MK-4827	Not approved	PARP 1,2	HER2 negative, BRCA mutation
Rucaparib/AG-014699	Not approved	PARP 1,2,3	TNBC and/or germline BRCA mutation, homologous recombination deficiency
Talazoparib/BMN-673	Approved	PARP 1,2	HER2 negative, germline BRCA mutation, stage I to III
Veliparib/ABT-888	Not approved	PARP 1,2	Triple-negative breast cancer, stage II to III
Pamiparib/AG-14361	Not approved	PARP 1	HER2 negative, germline BRCA mutation, stage II

## Data Availability

Not applicable.
